# Opioid use prior to elective surgery is strongly associated with persistent use following surgery: an analysis of 14 354 Medicare patients

**DOI:** 10.1111/ans.15492

**Published:** 2019-10-21

**Authors:** Max Catchpool, Josh Knight, Jesse T. Young, Philip Clarke, Michael J. Barrington, Peter F. M. Choong, Michelle M. Dowsey

**Affiliations:** ^1^ Centre for Health Policy, Melbourne School of Population and Global Health The University of Melbourne Melbourne Victoria Australia; ^2^ Department of Surgery The University of Melbourne, St Vincent's Hospital Melbourne Victoria Australia; ^3^ Justice Health Unit, Centre for Health Equity, Melbourne School of Population and Global Health The University of Melbourne Melbourne Victoria Australia; ^4^ Centre for Adolescent Health Murdoch Children's Research Institute Melbourne Victoria Australia; ^5^ Department of Anaesthesia and Acute Pain Medicine St Vincent's Hospital Melbourne Victoria Australia; ^6^ Melbourne Medical School, Faculty of Medicine, Dentistry and Health Sciences The University of Melbourne Melbourne Victoria Australia; ^7^ Department of Orthopaedics St Vincent's Hospital Melbourne Victoria Australia

**Keywords:** general surgery, orthopaedic surgery, urology

## Abstract

**Background:**

Persistent opioid use following total joint replacement (TJR) surgery is common; however, the association between pre‐surgical opioid use and surgery type has not been established. The objective of this study was to determine the association between pre‐surgery opioid use and persistent post‐surgery opioid use in TJR patients compared to other elective surgical patients.

**Methods:**

This is a retrospective cohort study, of univariate and multinomial logistic regression of linked, de‐identified Medicare Benefits Schedule and Pharmaceutical Benefits Schedule data, adjusted for perioperative opioid use, age and sex. Oral morphine equivalents daily doses (OMEDD) were calculated and opioid use was categorized into three mutually exclusive categories for each observation window: low (0–5 OMEDD), moderate (5–10 OMEDD), high (10+ OMEDD). Persistent opioid use was defined as opioid use between 180 and 270 days after the date of surgery.

**Results:**

Persistent opioid use was associated with older age, female gender and pre‐surgery opioid use. There was no increased risk for persistent opioid use for TJR patients compared to other surgical patients. The intensity of pre‐surgery opioid usage is strongly associated with persistent opioid use in all observed surgical patients.

**Conclusions:**

Our results suggest that many patients who use opioids prior to surgery will persist in their opioid use following surgery. No association was found between persistent opioid use and TJR surgery, but rather a risk reduction compared to other elective surgeries when associations with opioid use are controlled for. Primary care clinicians and surgeons should monitor the duration and dosage of perioperative opioid use.

## Graphical abstract

While pain relief is more commonly associated with total joint replacement patients than other surgical procedures it is not associated with an increased risk when pre‐surgical opioid use is taken into account. Primary care clinicians and surgeons should monitor the duration and dosage of pre‐surgery opioid use regardless of surgery procedure and conservative pre‐operative management of total joint replacement patients with opioids should be discouraged.

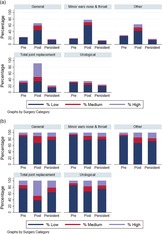

## Introduction

Total joint replacement (TJR) is a common surgical procedure for treating osteoarthritis of the hip and knee, a leading cause of pain and disability, and the 11th highest contributor to disability, globally.^1^ Due to concerns regarding the lifespan of current prostheses, patients are typically conservatively managed with the goal of extending the time until surgical intervention is required.^2^, ^3^ Typical conservative management pathways include medications for pain management, until their reduced effectiveness necessitate surgical intervention.

Guidelines for managing hip and knee osteoarthritis have previously recommended the use of opioids as a method of pain management.^2^, ^4^ In Australia, recommendations introduced in 2009 stated that opioids should be used in patients who have not responded or could not tolerate other analgesic medication, and in whom surgery was contraindicated or delayed.^4^ However, current Australian guidelines (2018),^3^ recommend against the use of opioids at any stage of osteoarthritis, which is anticipated to be concordant with pending updated American guidelines. This represents a shift in osteoarthritis management strategies due to growing public health concerns about potential harms of prescription opioid misuse and morbidity.

Post‐surgery, TJR patients are at higher risk of persistent opioid use compared to other frequently performed procedures.^5^ This increased risk has only been observed in opioid naïve patients however, which raises questions about the generalizability to an opioid‐exposed population, which is likely to be more common among a conservatively managed population. Prior research has observed that pre‐surgery opioid use is a risk factor for persistent opioid use following TJR;^6^ however, no study has simultaneously examined the association between pre‐surgery opioid use and persistent opioid use in TJR compared to other frequently performed elective procedures. Therefore, the relative importance of type of surgical procedure and pre‐opioid use on patterns of post‐surgical opioid use is yet to be established.

A greater understanding of the perioperative use of opioids in TJR patients is central to reducing persistent, contraindicated opioid use. Furthermore, these findings could inform future studies aimed at establishing the benefits and harms associated with the use of opioids in conservative management strategies, and optimum timing for TJR surgery. In this population‐based study, we sought to determine the association between pre‐surgery opioid use and persistent post‐surgery opioid use in TJR patients compared to other elective surgical patients.

## Methods

### Data set

A linked 10% sample (almost 3 million Australians) containing all federally funded medical and hospital services (Medicare Benefits Schedule (MBS))^7^ and pharmaceutical (Pharmaceutical Benefits Schedule (PBS)) claims data (i.e. medication dispensing records) for the period 1 April 2012 and 31 December 2014. Both claims datasets and patient information were deterministically linked to demographic data (year of birth and gender) using a unique identifier.

The MBS data set covered healthcare subsidized by the Australian Federal Government and the total fee charged for the surgical procedure. It contains all elective surgeries performed on patients in private hospitals, and those conducted on private patients in public hospitals. It does not capture public patients treated in public hospitals, who are funded through the State departments of health. The PBS data set captures all prescription medication subsidized by the Australian Federal government from 1 April 2012. Prior to this the government did not collect dispensing record information for medicines which fell under the co‐payment threshold.

Fifteen procedures with the longest elective surgery wait time were selected as candidates for the analysis based on the 2015 Australian Institute of Health and Welfare's report by procedure for public hospitals 2015.^8^ Cataract surgery was removed because it is typically an outpatient procedure with limited pain. Tonsillectomy was also removed from the analysis, as a procedure primarily undertaken in children. The remaining 13 procedures were classified based on the National Elective Surgery Urgency Categorisation Guidelines (2015)^9^ into related broader groups (Table 1). The Australian Classification of Health Interventions eighth edition^10^ was then used to identify the MBS codes (Appendix S1) associated with all surgical categories remaining. These MBS codes were then used to identify eligible individuals within the MBS data.

**Table 1:** Variable and categorizations of included surgical groups†

Subcategory‡	Analysis category§	Included in final analysis
Hip TJR	TJR	Yes
Knee TJR		Yes
Cholecystectomy	General	Yes
Haemorrhoidectomy		Yes
Inguinal herniorrhaphy		Yes
Cystoscopy	Urological	Yes
Prostatectomy		Yes
Myringoplasty	Minor ears nose and throat	Yes
Myringotomy		Yes
Septoplasty		Yes
Coronary artery bypass graft	Other	Yes
Hysterectomy		Yes
Varicose veins stripping and ligation		Yes
Cataract surgery	NA	No
Tonsillectomy	NA	No

† Fifteen candidate operation selected on the basis of length of elective surgery wait time (2015 Australian Institute of Health and Welfare data), the mapping onto the National Elective Surgery Urgency Categorisation Guidelines categories and the inclusion status in the current analysis.

‡ Based on the Australian Institute of Health and Welfare category.

§ Based on the National Elective Surgery Urgency Categorisation Guidelines.

TJR, total joint replacement.

To enable a 180‐day lead up and a 270‐day follow up period to establish whether opioids were used pre‐ and post‐surgery, we identified all first instances of elective surgery between 1 October 2012 and 5 April 2014 (herein referred to as the index surgery). If patients underwent more than one eligible procedure during the study period, we included only the first (index) procedure. We then identified the use of opioids within the sample based on dispensing information and using PBS item codes (Appendix S2). Pre‐surgery opioid use was defined as the period 180 days before the date of surgery. We defined post‐surgery opioid use as the 30‐day period from the date of the index surgery and persistent opioid use, our primary outcome, as between 180 and 270 days after the date of the index surgery.

To quantify the strength of opioid use, total daily oral morphine equivalents were calculated. Oral morphine equivalents are used to standardize the impact of a range of classes and doses of available opioids into a common unit of analysis allowing for direct comparison of a range of medication regimes.^11^ The type and strength of the opioid medication was multiplied by a conversion factor obtained from the Australian and New Zealand College of Anesthetists Faculty of Pain Management.^12^ We calculated oral morphine equivalents daily doses (OMEDD) by dividing total morphine equivalents dispensed by the number of days in our opioid use observation windows (i.e. pre‐surgery, post‐surgery and persistent periods), to allow a direct per day comparison across these observation windows. Using OMEDDs, we categorized opioid use into three mutually exclusive categories for each observation window, respectively: low (0–5 OMEDD), moderate (5–10 OMEDD) and high (10+ OMEDD).

### Statistical analysis

All analyses were performed on Stata Version 14.1 (StataCorp, College Station, TX, USA). We fit univariate and multivariate multinominal logistic regression models with robust standard errors to calculate the relative risk of persistent opioid use for the different opioid categories. The multivariate model was adjusted for covariates pre‐surgery opioid use, post‐surgery opioid use, age and gender with the dependant variable being persistent opioid use. Sensitivity analyses were undertaken to test for the importance of prior opioid use in the 180 days before surgery by restricting the analysis to those who were opioid naïve. A sensitivity analysis was also run restricting the population to those who were opioid exposed in the 180 days prior to surgery. To test for the importance of the OMEDD threshold a sensitivity analysis was undertaken with the range substantially widened (0, 0–10 OMEDD, 10–20 OMEDD and 20+ OMEDD). Finally, all individual who had a second eligible operation within the follow‐up time were removed from the population and the analysis undertaken. This is in contrast to the main analysis where these individuals were retained but only the first operation included in the analysis. All tests were two‐tailed, and significance was set at *P* < 0.05 We report this study according to the reporting of studies using observational routinely collected health data checklist.^13^

### Ethics approval

This study received ethics approval from the University of Melbourne Human Research Ethics Committee (No. 1749347).

## Results

The demographic profile and surgical category of 14 354 individuals who underwent at least one eligible elective surgery during the study period are described in Table 2.

**Table 2:** Demographics

	*n*	Age (years), mean (SD)	Female, *n* (%)
Total joint replacement†	4576	68.0 (10.0)	2504 (54.7)
General‡	3446	56.4 (16.1)	1091 (31.7)
Urological§	2868	61.8 (14.4)	1211 (42.2)
Minor ears, nose and throat¶	1945	42.4 (19.2)	925 (47.6)
Other††	1519	57.4 (14.2)	1053 (69.3)
Total	14 354	59.4 (16.5)	47.3%

† Hip replacement and knee replacement.

‡ Cholecystectomy, haemorrhoidectomy and inguinal herniorrhaphy.

§ Cystoscopy and prostatectomy.

¶ Myringoplasty, myringotomy and septoplasty.

†† Coronary artery bypass graft, hysterectomy, varicose veins stripping and ligation.

**Figure 1:**
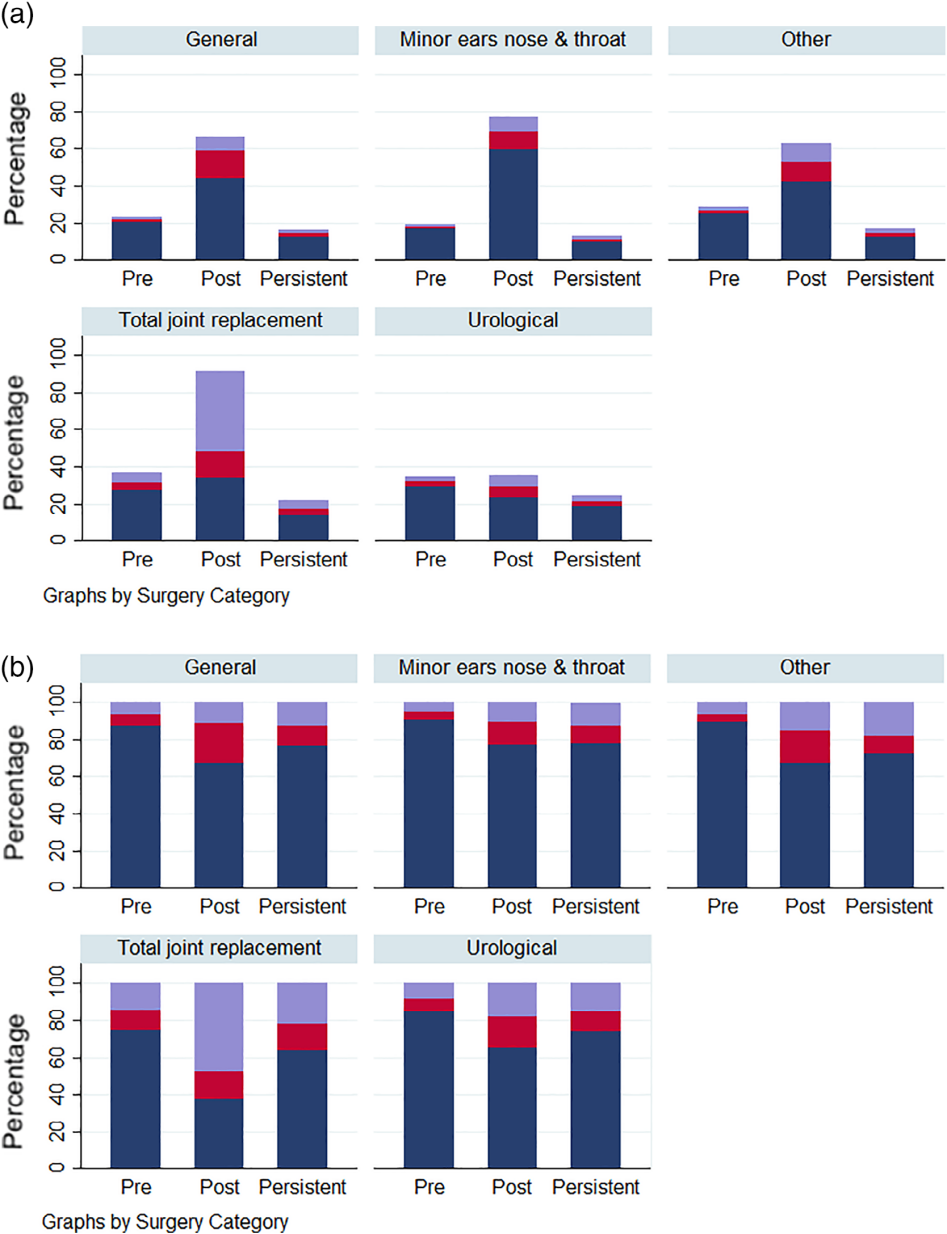
(a) Opioid use by surgical procedure and observation windows. (b) Intensity of opioid use given usage in the specified period. Description – low = 0–5 oral morphine equivalent daily dose (OMEDD), moderate = 5–10 OMEDD, high = 10+ OMEDD. Pre‐surgery opioid use = 180 days before date of surgery, post‐surgery opioid use = 30‐day period including and following date of surgery, persistent opioid use =180–270 days after date of surgery. Total joint replacement = hip replacement and knee replacement. General = cholecystectomy, haemorrhoidectomy and inguinal herniorrhaphy. Minor ears nose and throat = myringoplasty, myringotomy and septoplasty. Urological = cystoscopy and prostatectomy. Other = coronary artery bypass graft, hysterectomy and varicose veins stripping and ligation. (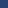) % Low; (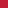) % medium; (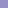) % high.

Figure 1a shows the proportion of patients using the different levels of opioids across the observation windows (pre‐surgery, post‐surgery and persistent periods) and the different surgical procedures. Pre‐surgery opioid use ranged from 19.7% for minor ears, nose and throat (ENT) to 36.9% for TJR. Post‐surgery opioid use ranged from 35.6% for urological to 91.7% for TJR. The proportion of persistent opioid use ranged from 12.6% for ENT to 24.8% for urological surgery. Figure 1b shows the proportion of opioid usage across the oral morphine equivalent thresholds for those who were dispensed opioids in the observation windows. Further detail on units dispensed and total oral morphine equivalents' across the three observation windows is provided in Appendix S3. Compared to other surgical categories TJR patients had a higher intensity of opioid use.

On univariate analysis age, gender, pre‐surgery opioid use, post‐surgery opioid use and surgical category were significantly associated with persistent opioid use (Appendix S4). From the multinomial logistic regression analysis age and gender remained significant (Table 3). After adjustment for model covariates, pre‐surgery opioid use remained significant but there was a reduction in effect size across each persistent opioid use level. For the low post‐surgery opioid use group, the association between persistent moderate and high opioid use was no longer significant. The association between persistent moderate opioid use and moderate post‐surgery opioid use was significant.

**Table 3:** Multinomial logistic regression†

	Persistent opioid use (180–270 days after date of surgery) (reference = none)		
	Low	Moderate	High
Age (per 10 years)	**1.1 (1.1–1.8)**	**1.4 (1.2–1.5)**	**1.3 (1.2–1.4)**
Male (reference = female)	**0.7 (0.6–0.8)**	**0.7 (0.5–0.9)**	**0.7 (0.5–0.9)**
Pre‐surgery opioid use (180‐day before date of surgery)			
None (reference)	1	1	1
Low (0–5 OMEDD)	**1.6 (1.5–1.8)**	**2.8 (2.1–3.7)**	**3.0 (2.2–4.0)**
Moderate (5–10 OMEDD)	**3.3 (2.5–4.5)**	**26.7 (18.9–37.7)**	**13.2 (8.6–20.1)**
High (10+ OMEDD)	**4.4 (3.1–6.1)**	**15.6 (9.8–25.0)**	**97.8 (70.8–135.0)**
Post‐surgery opioid use (30‐day period including and following date of surgery)			
None (reference)	1	1	1
Low (0–5 OMEDD)	**0.5 (0.5–0.6)**	0.7 (0.5–1.0)	0.9 (0.6–1.2)
Moderate (5–10 OMEDD)	**0.6 (0.5–0.7)**	1.4 (0.9–2.1)	**1.9 (1.2–2.9)**
High (10+ OMEDD)	**0.6 (0.5–0.7)**	**1.7 (1.1–2.5)**	**3.9 (2.7–5.7)**
Surgical category			
Total joint replacement (reference)‡	1	1	1
Minor ears, nose and throat§	0.9 (0.8–1.2)	1.5 (0.9–2.5)	**1.9 (1.2–3.0)**
General¶	1.0 (0.9–1.2)	**1.5 (1.0–2.1)**	**1.7 (1.2–2.4)**
Urological††	**1.2 (1.0–1.4)**	**1.7 (1.2–2.4)**	**2.5 (1.8–3.5)**
Other‡‡	0.9 (0.7–1.0)	1.1 (0.7–1.8)	**2.3 (1.5–3.4)**

† Bold values represent significance at *P* < 0.05.

‡ Hip replacement and knee replacement.

§ Myringoplasty, myringotomy and septoplasty.

¶ Cholecystectomy, haemorrhoidectomy and inguinal herniorrhaphy.

†† Cystoscopy and prostatectomy.

‡‡ Coronary artery bypass graft, hysterectomy, varicose veins stripping and ligation. OMEDD, oral morphine equivalent daily dose.

After adjustment for model covariates, low persistent opioid use was not associated with ENT and general surgeries. Moderate persistent opioid use was no longer associated with ENT and other surgeries. General surgery remained significant when compared to TJR; however, the direction of the relative risk changed from a risk reduction to a relative risk increase. Compared to TJR, the relative risk for a positive association between urological surgery and persistent opioid use became statistically significant. All surgical types were associated with a significant increased relative risk of high persistent opioid use when compared to TJR (Table 3).

Sensitivity analyses largely supported our primary analysis. When the sample was restricted to opioid‐naïve individual's post‐surgery opioid use was associated with reduced persistent low and moderate opioid use. No association was observed between surgical category and persistent opioid use in opioid‐naïve individuals (Appendix S5.1). To test the importance of opioid use in the lead up to surgery we also performed sensitivity analyses restricting the sample to individuals who were opioid users in the 180 days prior to surgery. Contrary to primary analyses, positive associations were observed across all levels of opioids for post‐surgery opioid use. A larger effect size was also observed across all surgery categories for persistent moderate and high opioid use when compared to TJR as the reference category (Appendix S5.2). To assess the effect of multiple surgeries within the follow‐up period, we repeated the analyses and restricted it to individuals who only underwent one surgery during follow‐up (*n* = 13 847), no differences were observed from the main analysis (Appendix S5.3). The effect size of coefficients remained similar when the analyses were repeated using four different OMEDD thresholds (0, 0–10 OMEDD, 10–20 OMEDD and 20+ OMEDD) (Appendix S5.4).

## Discussion

In this retrospective cohort study of 14 354 patients who underwent an elective surgical procedure there was evidence of substantial persistent opioid use (between 12.6% and 24.8%) across all surgical categories. Persistent opioid use was associated with older age, female gender and pre‐surgery opioid use. Controlling for these variables, TJR was associated with either the same, or lower risk, of persistent opioid use when compared to other surgical categories. Our results indicate that the prescribing practices associated with patients, rather than the operation itself, are the key contributing factors to persistent opioid use. This was supported by sensitivity analysis which found no significant relationship between surgical category and persistent opioid use among those who were opioid naïve during the pre‐surgical period.

### Implications for clinical practice

Our results suggest that primary care clinicians and surgeons should monitor the duration and dosage of perioperative opioid use, in line with current guidelines.^3^ The successful management of patients require a balancing of benefits between conservative management with the benefits of minimization of opioid use with the potential consequence of reduced time to surgery.

Conservative management of patients with osteoarthritis is based on well‐founded concerns which include the lifespan of implants. The prevalence of revision surgery after TJR is 12% after 10 years,^14^ with revision surgery also less successful than primary TJR.^15^ Concerns over the lifespan of implants and the potential consequences of revision surgery often result in clinicians using methods such as physiotherapy and pharmaceutical based pain management to delay the time until a TJR is required.^2^, ^4^ While pain relief is a key feature of appropriate conservative management,^2^ the role of opioids in this process is contraindicated.^3^

Our findings pre‐date current Royal Australian College of General Practitioners Guidelines for the management of osteoarthritis which recommend against opioid use. The impact of these guidelines on the prescribing practices of general practitioners, where the majority of prescribing in Australia occurs, has yet to be elucidated. Following the release of the 2009 guidelines an increase in prescribing of potent opioids to future TJR patients was observed^16^ for reasons that remain unclear. Utilization of non‐pharmacological treatments including physiotherapy and weight loss as first‐line strategies are low compared to pharmacological management rates for osteoarthritis.^17^ Lack of resources and out‐of‐pocket costs associated with community‐based interventions may affect participation. Inability to pay has been cited as a deterrent by primary care physicians in referring patients with osteoarthritis to physiotherapy.^18^

Given the continued role of opioids in the management of pain in the TJR population the benefits of conservative management should be assessed against the potential harms of prolonged opioid analgesic use which include tolerance, dependence, opioid‐induced hyperalgesia, misuse, abuse and risk of accidental overdose.^19^ Hospitalizations and overdose deaths from prescribed opioids have increased rapidly in Australia and internationally,^20^, ^21^ the association between opioid use and these harms is both chronicity‐ and dose‐dependent.^19^

While our findings broadly align with the international literature,^5^ direct comparisons of our results with others is difficult due to differences in the definition of time periods, patient populations, application of the data and whether opioid naïve cohorts are being observed. Collaborative efforts on an international scale are warranted to address the opioid crisis, given the global nature of the problem.

### Strength and limitations

A strength of this study is the generation of findings from a representative sample from national level, routinely collected administrative data. This high‐quality data is more accurately able to identify the opioid consumption of each patient than patient‐reported opioid use histories.^22^ We were also able to compare multiple surgery types. A range of studies exist which have evaluated opioid use for TJR patients in isolation, but few have made comparisons across surgery types; we were unable to identify a study where pre‐surgery opioid users were analysed.^23^

Several limitations are worth noting. The use of an administrative data set limited the number of covariates we could include in the regression model and we are unable to adjust for unmeasured confounders. The nature of the data set also means that we can only identify correlations rather than causal factors. The inability to identify, and adjust for, comorbidities might confound the association between operative incidence and persistent opioid use. The surgical categories used in this analysis did not allow us to separate opioid use according to, for example minimally invasive versus open surgery. This level of granularity is more suited to a clinical trial, that would likely have a much smaller denominator, or a registry‐based study, which, unlike the current data, are typically focused on a single surgical category (i.e. orthopaedics).

Finally, this data represents privately insured patients who received an elective procedure in a public or private hospital. There has been an increase in the share of TJRs provided in private hospitals, from around 55% in 2000 to 70% in 2014.^24^ However, lower socioeconomic status has previously been identified as a risk factor for prolonged opioid use,^25^ therefore, our findings from this private patient cohort are likely a conservative estimate of the true effect.

## Conclusions

In summary, our results suggest that many patients who use opioids prior to surgery will persist in their opioid use post‐surgery. No association was found between persistent opioid use and TJR, but rather a risk reduction for high intensity opioid use compared to other elective surgeries when associations with pre‐surgery opioid use are controlled for. While we have confidence in this data there are limitations in assigning causality and controlling for potential confounding when analysing observational data. A study with random assignment to a conservative management or accelerated surgical management pathway would allow for a more in‐depth understanding of relative benefits for the alternate methods of management. While the observation nature of the data limits causal inference the strong association between pre‐surgery opioid use and persistent opioid use suggest that further study of this association is warranted.

## Conflicts of interest

None declared.

## Supporting information


**Appendix** **S1**. Medicare Benefits Schedule surgery item numbers.

**Appendix** **S2**. Pharmaceutical Benefits Schedule opioid item numbers.

**Appendix** **S3**. Opioid units dispensed and total oral morphine equivalents (OME's).

**Appendix** **S4**. Univariate Logistic Regression.

**Appendix** **S5**. Sensitivity Analysis.
