# Supplementary material for: Opioid use prior to elective surgery is strongly associated with persistent use following surgery: an analysis of 14 354 Medicare patients
Source: ANZ J Surg. 2019 Oct 21;89(11):1410–6. doi: 10.1111/ans.15492 (PMC6900005; doi:10.1111/ans.15492)
Supplement: Supplementary file 1 — Appendix S1. Medicare Benefits Schedule surgery item numbers. [file ANS-89-1410-s001.docx]

**Appendix File S1:** **Medicare Benefits Schedule surgery item numbers**

| Replacement of Knee | 49518, 49519, 49521, 49524, 49527, 49530, 49533, 49554, 49534 |
| --- | --- |
| Replacement of Hip | 49318, 49319, 49324, 49327, 49330, 49333, 49345, 49339, 49342 |
| Cholecystectomy | 30443, 30454, 30455, 30448, 30449 |
| Coronary artery bypass graft | 38497, 39497, 38500, 38503, 38500 |
| Cystoscopy | 36812, 36812, 36836 |
| Haemorrhoidectomy | 32138, 32132, 32135 |
| Hysterectomy | 35653, 35661, 35670, 35667, 35664, 35657, 35756, 90450, 90448 |
| Inguinal herniorrhaphy | 30614, 30615, 30609 |
| Myringoplasty | 41527, 41530, 41533, 41542, 41635 |
| Myringotomy | 41626, 41632 |
| Prostatectomy | 37203, 37207, 37200, 37209, 90407, 37201, 37224, 37209, 37210, 37211, 90408 |
| Septoplasty | 41671 |
| Varicose veins stripping and ligation | 32508, 32511, 32504, 32507, 32514 |
