# Supplementary material for: Opioid use prior to elective surgery is strongly associated with persistent use following surgery: an analysis of 14 354 Medicare patients
Source: ANZ J Surg. 2019 Oct 21;89(11):1410–6. doi: 10.1111/ans.15492 (PMC6900005; doi:10.1111/ans.15492)
Supplement: Supplementary file 2 — Appendix S2. Pharmaceutical Benefits Schedule opioid item numbers. [file ANS-89-1410-s002.docx]

**Appendix File S2: Pharmaceutical Benefits Schedule opioid item numbers**

| 1607N, 1644M, 1645N, 1646P, 1647Q, 1653B, 1654C, 1655D, 1656E, 2122Q, 2123R, 2124T, 2839K, 2840L, 2841M, 3479D, 3480E, 4349X, 5163R, 5168B, 5237P, 5238Q, 5239R, 5391R, 5392T, 5393W, 5394X, 5395Y, 5396B,8035X, 8146R, 8305D, 8306E, 8349K, 8453X, 8454Y, 8489T, 8490W, 8491X, 8492Y, 8493B, 8494C, 8669G, 8670H, 5116G, 8420E, 8421F, 8422G, 8423H, 8424J, 8541M, 8542N, 8543P, 9299K, 9406C, 9407D, 9408E, 9409F, 2481N, 2622B, 5190E, 5191F, 5194J, 5195K, 5197M, 8385H, 8386J, 8387K, 8388L, 8464L, 8501K, 8502L, 8644Y, 9399Q, 9400R, 8000C, 8934F, 8935G, 8936H, 1215Y, 3316M, 4170L, 4171M, 8785J, 5265D, 5277R, 5278T, 5279W, 5280X, 5401G, 5402H, 5403J, 5404K, 5405L, 5406M, 5407N, 5408P, 5409Q, 5410R, 5411T, 5412W, 5437E, 5438F, 5439G, 5440H, 5441J, 8878G, 8891Y, 8892B, 8893C, 8894D, 8865N, 8866P, 8867Q, 2527B, 3484J, 5231H, 5232J, 8455B, 8523N, 8524P**,** 8525Q, 8582Q, 8611F, 8843K, 10091D, 10092E, 10094G, 10096J, 10100N |
| --- |
