# Supplementary material for: Opioid use prior to elective surgery is strongly associated with persistent use following surgery: an analysis of 14 354 Medicare patients
Source: ANZ J Surg. 2019 Oct 21;89(11):1410–6. doi: 10.1111/ans.15492 (PMC6900005; doi:10.1111/ans.15492)
Supplement: Supplementary file 4 — Appendix S4. Univariate Logistic Regression. [file ANS-89-1410-s004.docx]

**Appendix File S4: Univariate** **Logistic Regression**

|  | | Persistent Opioid Use (reference = none) | | | |
| --- | --- | --- | --- | --- | --- |
|  | | Low | | Moderate | High |
| Age (per 10 years) | | **1.2**  **(1.1 – 1.2)** | | **1.4**  **(1.3 – 1.6)** | **1.4**  **(1.3 – 1.6)** |
| Male (Reference = Female) | | **0.7**  **(0.7 – 0.8)** | | **0.7**  **(0.5 – 0.8)** | **0.6**  **(0.5 – 0.7)** |
| Pre-surgery opioid use | None  (reference) | 1 | | 1 | 1 |
|  | Low | **1.9**  **(1.7 – 2.1)** | | **3.2**  **(2.4 – 4.2)** | **3.3**  **(2.5 – 4.4)** |
|  | Moderate | **3.5**  **(2.6 – 4.6)** | | **39.2**  **(28.1 – 54.7)** | **22.3**  **(14.9 – 33.4)** |
|  | High | **4.4**  **(3.2 – 6.0)** | | **23.8**  **(15.3 – 37.1)** | **201.3**  **(148.4 – 273.0)** |
| Post-surgery opioid use | None  (reference) | 1 | | 1 | 1 |
|  | Low | **0.5**  **(0.4 – 0.5)** | | **0.6**  **(0.4 – 0.8)** | **0.7**  **(0.5 – 0.9)** |
|  | Moderate | **0.6**  **(0.5 – 0.7)** | | **1.6**  **(1.1 – 2.2)** | **1.9**  **(1.3 – 2.7)** |
|  | High | **0.7**  **(0.6 – 0.7)** | | **2.3**  **(1.7 – 3.1)** | **7.8**  **(6.0 – 10.3)** |
| Surgical categories | Total Joint Replacement  (reference) | 1 | | 1 | 1 |
|  | Minor ears, nose and throat | **0.6**  **(0.5 – 0.8)** | | **0.4**  **(0.2 – 0.6)** | **0.3**  **(0.2 – 0.4)** |
|  | General | **0.8**  **(0.7 – 1.0)** | | **0.6**  **(0.4 – 0.8)** | **0.4**  **(0.3 – 0.5)** |
|  | Urological | **1.4**  **(1.2 – 1.6)** | | 0.9  (0.7 – 1.2) | 0.8  (0.7 – 1.0) |
|  | Other | 0.9  (0.7 – 1.0) | | **0.5**  **(0.3 – 0.8)** | **0.6**  **(0.5 – 0.9)** |
| ^Low = 0-5 Oral morphine equivalent daily dose (OMEDD)^  ^Moderate = 5-10 OMEDD^  ^High = 10+ OMEDD^  ^Pre-surgery opioid use = 180-days before date of surgery^  ^Post-surgery opioid use = 30-day period including and following date of surgery^  ^Persistent opioid use =180-270 days after date of surgery^ | | | ^Total Joint Replacement = Hip replacement, Knee replacement^  ^General = Cholecystectomy, Haemorrhoidectomy, Inguinal herniorrhaphy^  ^Minor ears nose & throat = Myringoplasty, Septoplasty^  ^Urological = Cystoscopy, Prostatectomy,^  ^Other = Coronary artery bypass graft, Hysterectomy, Varicose veins stripping and ligation^  ^Bold values represent significance at P< 0.05.^ | | |
