# Supplementary material for: Opioid use prior to elective surgery is strongly associated with persistent use following surgery: an analysis of 14 354 Medicare patients
Source: ANZ J Surg. 2019 Oct 21;89(11):1410–6. doi: 10.1111/ans.15492 (PMC6900005; doi:10.1111/ans.15492)
Supplement: Supplementary file 5 — Appendix S5. Sensitivity Analysis. [file ANS-89-1410-s005.docx]

**Appendix File S5: Sensitivity Analysis**

**Sensitivity Analysis – 5.1:** **Analysis restricted to individuals who were opioid-naïve**

|  | | Persistent Opioid Use (reference = none) | | |
| --- | --- | --- | --- | --- |
|  | | Low | Moderate | High |
| Age (per 10 years) | | **1.1**  **1.0 - 1.1** | **1.3**  **1.0 - 1.5** | **1.5**  **1.2 - 1.7** |
| Male (Reference = Female) | | **0.7**  **0.6 - 0.8** | 0.8  0.5 - 1.1 | **0.7**  **0.4 – 1.0** |
| Post-surgery opioid filling | None  (reference) | 1 | 1 | 1 |
|  | Low | **0.2**  **0.2 - 0.3** | **0.2**  **0.1 - 0.4** | **0.3**  **0.1 - 0.5** |
|  | Moderate | **0.3**  **0.2 - 0.3** | **0.3**  **0.1 - 0.6** | 0.6  0.3 - 1.2 |
|  | High | **0.3**  **0.2 - 0.3** | **0.5**  **0.3 – 1.0** | 0.7  0.4 - 1.4 |
| Surgical category | Total Joint Replacement  (reference) | 1 | 1 | 1 |
|  | Minor ears, nose and throat | 0.8  0.6 - 1.0 | 0.8  0.4 - 1.7 | 0.8  0.4 – 2.0 |
|  | General | 0.9  0.7 - 1.1 | 0.8  0.4 – 1.5 | 0.6  0.3 - 1.2 |
|  | Urological | 0.9  0.7 - 1.1 | 0.6  0.3 - 1.2 | 0.8  0.4 - 1.5 |
|  | Other | 0.8  0.6 - 1.1 | 0.4  0.2 - 1.1 | 0.7  0.4 - 1.5 |

| ^Low = 0-5 OMEDD^  ^Moderate = 5-10 OMEDD^  ^High = 10+ OMEDD^  ^Post-surgery opioid use = 30-day period including and following date of surgery^  ^Persistent opioid use =180-270 days after date of surgery^ | ^Total Joint Replacement = Hip replacement, Knee replacement^  ^General = Cholecystectomy, Haemorrhoidectomy, Inguinal herniorrhaphy^  ^Minor ears nose & throat = Myringoplasty, Septoplasty^  ^Urological = Cystoscopy, Prostatectomy,^  ^Other = Coronary artery bypass graft, Hysterectomy, Varicose veins stripping and ligation^  ^Bold values represent significance at P< 0.05^ |
| --- | --- |

**Sensitivity Analysis – 5.2:** **Analysis restricted to individuals who were dispensed opioid medication in the pre-surgery period**

|  | | Persistent Opioid Use (reference = none) | | |
| --- | --- | --- | --- | --- |
|  | | Low | Moderate | High |
| Age (per 10 years) | | **1.2**  **(1.1 - 1.30)** | **1.3**  **(1.2 - 1.5)** | **1.2**  **(1.0 - 1.3)** |
| Male (Reference = Female) | | **0.8**  **(0.6 - 0.9)** | **0.7**  **(0.5 - 0.9)** | 0.8  (0.6 - 1.1) |
| Pre-surgery opioid filling | Low  (reference) | 1 | 1 | 1 |
|  | Moderate | **1.5**  **(1.1 - 2.0)** | **7.9**  **(5.5 – 11.4)** | **3.4**  **(2.0 - 5.6)** |
|  | High | **1.9**  **(1.4 - 2.7)** | **4.7**  **(2.7 - 8.1)** | **24.9**  **(16.8 - 36.9)** |
| Post-surgery opioid filling | None  (reference) | 1 | 1 | 1 |
|  | Low | **2.3**  **(1.8 - 2.9)** | **2.6**  **(1.5 - 4.5)** | **2.7**  **(1.4 - 5.2)** |
|  | Moderate | **2.5**  **(1.9 - 3.4)** | **4.4**  **(2.4 - 8.0)** | **7.6**  **(3.9 - 14.9)** |
|  | High | **2.4**  **(1.8 - 3.2)** | **5.4**  **(3.1 - 9.6)** | **15.3**  **(8.3 - 28.10** |
| Surgical category | Total Joint Replacement  (reference) | 1 | 1 | 1 |
|  | Minor ears, nose and throat | **1.4**  **(1.0 – 1.9)** | **2.9**  **(1.4 - 5.8)** | 1.9  (0.9 - 4.1) |
|  | General | 1.3  (1.0 - 1.6) | **2.3**  **(1.4 - 3.8)** | **2.8**  **(1.7 - 4.6)** |
|  | Urological | **1.7**  **(1.3 - 2.3)** | **2.8**  **(1.7 - 4.5)** | **4.0**  **(2.6 - 6.2)** |
|  | Other | 1.0  (0.7 - 1.4) | **2.4**  **(1.3 - 4.5)** | **4.6**  **(2.6 - 8.1)** |

| ^Low = 0-5 OMEDD^  ^Moderate = 5-10 OMEDD^  ^High = 10+ OMEDD^  ^Pre-surgery opioid use = 180-days before date of surgery^  ^Post-surgery opioid use = 30-day period including and following date of surgery^  ^Persistent opioid use =180-270 days after date of surgery^ | ^Total Joint Replacement = Hip replacement, Knee replacement^  ^General = Cholecystectomy, Haemorrhoidectomy, Inguinal herniorrhaphy^  ^Minor ears nose & throat = Myringoplasty, Septoplasty^  ^Urological = Cystoscopy, Prostatectomy,^  ^Other = Coronary artery bypass graft, Hysterectomy, Varicose veins stripping and ligation^  ^Bold values represent significance at P< 0.05^ |
| --- | --- |

**Sensitivity Analysis – 5.3: Analysis restricted to individuals who only underwent one surgery during follow up**

|  | | Persistent Opioid Use (reference = none) | | | |
| --- | --- | --- | --- | --- | --- |
|  | | Low | | Moderate | High |
| Age (per 10 years) | | **1.1**  **1.1 - 1.2** | | **1.4**  **(1.2 – 1.5)** | **1.3**  **(1.2 – 1.4)** |
| Male (Reference = Female) | | **0.7**  **(0.6 – 0.8)** | | **0.7**  **(0.5 – 0.9)** | **0.7**  **(0.5 – 0.9)** |
| Pre-surgery opioid use | None  (reference) | 1 | | 1 | 1 |
|  | Low | **1.6**  **(1.5 – 1.8)** | | **2.8**  **(2.1 – 3.7)** | **3.0**  **(2.2 – 4.0)** |
|  | Moderate | **3.3**  **(2.5 – 4.5)** | | **26.7**  **(18.9 – 37.7)** | **13.2**  **(8.6 – 20.1)** |
|  | High | **4.4**  **(3.1 – 6.1)** | | **15.6**  **(9.8 – 25.0)** | **97.8**  **(70.8 – 135.0)** |
| Post-surgery opioid use | None  (reference) | 1 | | 1 | 1 |
|  | Low | **0.5**  **(0.5 – 0.6)** | | 0.7  (0.5 – 1.0) | 0.9  (0.6 – 1.3) |
|  | Moderate | **0.6**  **(0.5 – 0.7)** | | 1.4  (1.0 – 2.1) | **1.9**  **(1.2 – 2.9)** |
|  | High | **0.6**  **(0.5 – 0.7)** | | **1.7**  **(1.1 – 2.5)** | **3.9**  **(2.7 – 5.7)** |
| Surgical category | Total Joint Replacement  (reference) | 1 | | 1 | 1 |
|  | Minor ears, nose and throat | 0.9  (0.8 – 1.2) | | 1.5  (0.9 – 2.5) | **1.9**  **(1.2 – 3.0)** |
|  | General | 1.0  (0.9 – 1.2) | | **1.5**  **(1.0 – 2.1)** | **1.7**  **(1.2 – 2.4)** |
|  | Urological | **1.2**  **(1.0 – 1.4)** | | **1.7**  **(1.2 – 2.4)** | **2.5**  **(1.8 – 3.5)** |
|  | Other | 0.9  (0.7 – 1.0) | | 1.1  (0.7 – 1.8) | **2.3**  **(1.5 – 3.4)** |
| ^Low = 0-5 OMEDD^  ^Moderate = 5-10 OMEDD^  ^High = 10+ OMEDD^  ^Pre-surgery opioid use = 180-days before date of surgery^  ^Post-surgery opioid use = 30-day period including and following date of surgery^  ^Persistent opioid use =180-270 days after date of surgery^ | | | ^Total Joint Replacement = Hip replacement, Knee replacement^  ^General = Cholecystectomy, Haemorrhoidectomy, Inguinal herniorrhaphy^  ^Minor ears nose & throat = Myringoplasty, Septoplasty^  ^Urological = Cystoscopy, Prostatectomy,^  ^Other = Coronary artery bypass graft, Hysterectomy, Varicose veins stripping and ligation^  ^Bold values represent significance at P< 0.05^ | | |

**Sensitivity Analysis – 5.4: Widened OMEDD thresholds**

|  | | Persistent Opioid Use (reference = none) | | | |
| --- | --- | --- | --- | --- | --- |
|  | | Low | | Moderate | High |
| Age (per 10 years) | | **1.2**  **(1.1 – 1.2)** | | **1.4**  **(1.2 – 1.6)** | **1.2**  **(1.1 – 1.4)** |
| Male (Reference = Female) | | **0.7**  **(0.6 – 0.8)** | | **0.5**  **(0.4 – 0.7)** | **0.9**  **(0.6 - 1.2)** |
| Pre-surgery opioid use | None  (reference) | 1 | | 1 | 1 |
|  | Low | **2.0**  **(1.8 – 2.2)** | | **3.0**  **(2.1 – 4.3)** | **4.7**  **(3.1 – 7.2)** |
|  | Moderate | **6.4**  **(4.5 – 9.3)** | | **68.3**  **(42.6 – 109.6)** | **46.6**  **(26.5 – 82.1)** |
|  | High | **3.7**  **(2.3 – 6.1)** | | **38.1**  **(20.9 – 69.2)** | **191.4**  **(112.9 -324.3)** |
| Post-surgery opioid use | None  (reference) | 1 | | 1 | 1 |
|  | Low | **0.6**  **(0.5 – 0.7)** | | 1.3  (0.8 – 2.0) | 1.2  (0.7 – 2.0) |
|  | Moderate | **0.7**  **(0.6 – 0.9)** | | **3.5**  **(2.1 – 2.8)** | **2.2**  **(1.2 – 4.3)** |
|  | High | 0.8  (0.7 – 1.0) | | **4.9**  **(2.8 – 8.3)** | **8.7**  **(4.9 – 15.3)** |
| Surgical category | Total Joint Replacement  (reference) | 1 | | 1 | 1 |
|  | Minor ears, nose and throat | 1.0  (0.8 – 1.2) | | **1.9**  **(1.0 – 3.5)** | 1.7  (0.9 -3.4) |
|  | General | 1.1  (0.9 – 1.3) | | **1.6**  **(1.0 – 2.7)** | **1.8**  **(1.1 – 2.9)** |
|  | Urological | **1.3**  **(1.1 – 1.5)** | | **2.6**  **(1.7 – 3.8)** | **2.7**  **(1.7 – 4.3)** |
|  | Other | 0.9  (0.7 – 1.1) | | **1.8**  **(1.0 – 3.1)** | **2.9**  **(1.6 – 5.0)** |
| ^Low = 0-5 OMEDD^  ^Moderate = 5-10 OMEDD^  ^High = 10+ OMEDD^  ^Pre-surgery opioid use = 180-days before date of surgery^  ^Post-surgery opioid use = 30-day period including and following date of surgery^  ^Persistent opioid use =180-270 days after date of surgery^ | | | ^Total Joint Replacement = Hip replacement, Knee replacement^  ^General = Cholecystectomy, Haemorrhoidectomy, Inguinal herniorrhaphy^  ^Minor ears nose & throat = Myringoplasty, Septoplasty^  ^Urological = Cystoscopy, Prostatectomy,^  ^Other = Coronary artery bypass graft, Hysterectomy, Varicose veins stripping and ligation^  ^Bold values represent significance at P< 0.05^ | | |
